# Novel roles for protein disulphide isomerase in disease states: a double edged sword?

**DOI:** 10.3389/fcell.2015.00030

**Published:** 2015-05-21

**Authors:** Sonam Parakh, Julie D. Atkin

**Affiliations:** ^1^Department of Biomedical Sciences, Faculty of Medicine and Health Sciences, Macquarie UniversitySydney, NSW, Australia; ^2^Department of Biochemistry, La Trobe Institute for Molecular Science, La Trobe UniversityBundoora, VIC, Australia

**Keywords:** protein disulfide isomerase family, neurodegnerative diseases, protein chaperones, post-translational modifications, cancer, amyotrophic lateral sclerosis

## Abstract

Protein disulphide isomerase (PDI) is a multifunctional redox chaperone of the endoplasmic reticulum (ER). Since it was first discovered 40 years ago the functions ascribed to PDI have evolved significantly and recent studies have recognized its distinct functions, with adverse as well as protective effects in disease. Furthermore, post translational modifications of PDI abrogate its normal functional roles in specific disease states. This review focusses on recent studies that have identified novel functions for PDI relevant to specific diseases.

## Introduction

Protein disulphide isomerase (PDI) was the first folding catalyst isolated from rat liver (Goldberger et al., 1963) and it is found abundantly in many tissues, accounting for 0.8% of total cellular protein (Freedman et al., 1994). PDI is induced during endoplasmic reticulum (ER) stress (Wilkinson and Gilbert, 2004) and it serves as a vital cellular defense against general protein misfolding via its chaperone activity. It is also responsible for the isomerization, formation, and rearrangement of protein disulphide bonds, thereby providing another mechanism by which native protein conformation is maintained. Disulphide bonds play an important role in the folding and stability of proteins and they are present in more than 30% of all human proteins that traverse the secretory pathway (Fewell et al., 2001). Since most cellular compartments are reducing environments, protein disulphide bonds are usually unstable in the cytosol, although there are exceptions (Frand et al., 2000). PDI assists in redox protein folding, involving oxidation, multiple intramolecular thiol-disulphide exchanges, and isomerization (reduction) activities and it is highly specific in its interaction with different substrates. Whilst PDI is considered to be resident primarily within the ER, nonetheless it has been detected in many other diverse cellular locations, including the cell surface, cytosol, mitochondria, and extracellular matrix (Turano et al., 2002). However, the mechanism by which PDI escapes from the ER is still unclear. PDI is also present in the extracellular medium where it facilitates protein folding and reduces protein aggregation (Delom et al., 2001). Furthermore, specific functions of cell surface PDI have been identified in hepatocytes, platelets, and endothelial cells (Turano et al., 2002). This review focusses on recent advances recognizing the versatile roles of PDI in normal cellular function and also in disease states. These studies highlight novel therapeutic possibilities based on the functional properties of PDI.

## Structure and superfamily of PDI

PDI is a soluble 55-kDa protein that is the prototype of the PDI family of proteins which all contain the thioredoxin-like βαβαβαββα domain (Kemmink et al., 1997). Thioredoxins are a class of oxidoreductase enzymes containing a dithiol-disulphide active site that are involved in redox signaling (Moran et al., 2001). Besides PDI, 21 more family members have been described (Kozlov et al., 2010). However, the enzymatic properties of these proteins differ in their redox potential and hence substrate specificity (Jessop et al., 2009), the sequence of their active site and the pKa of the active site cysteine residues (Ellgaard and Ruddock, 2005). They are primarily localized in the ER where they maintain an oxidative environment and thereby contribute to ER homeostasis (Anelli et al., 2002).

**Figure 1:**
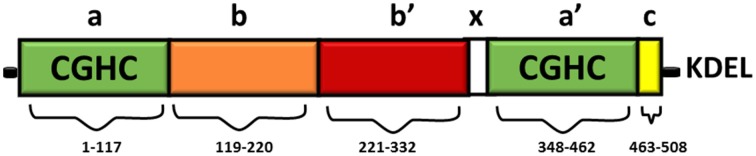
**Domain structure of PDI**. The thioredoxin-like domains are shown in green, representing the catalytically active domains *a* and *a'*. The catalytically inactive *b* domain and *b'* domains are illustrated in orange and red respectively. The linker region x (shown in white) is responsible for the U shape structure of PDI. The C terminus is illustrated in yellow, followed by an ER retrieval signal, KDEL.

Full length PDI contains 508 amino acids and consists of four domains namely *a, b, b', a'* (Figure 1). The homologous *a* and *a'* domains share 47% similarity and contain the active site, CGHC (Kemmink et al., 1996). The active site cysteine residues interact with the thiol group of a newly synthesized substrate, thus mediating the formation and isomerization of protein disulphide bonds (Gilbert, 1998). The intermediate *b* and *b'* domains are 28% identical and they assist in the binding of protein substrates but they lack the catalytically active cysteine residues (Gruber et al., 2006). PDI also contains a *x* linker region and an acidic C terminus containing a KDEL-ER retrieval sequence (Darby et al., 1996). Whilst the three dimensional structure of human PDI is still under investigation, the structures of each single thioredoxin domain (Nguyen et al., 2008) and the domain combinations *bb'c* (Denisov et al., 2009) and *bb'cxac* (Wang et al., 2012a) have been determined. However, the structure of yeast PDI has been solved (Tian et al., 2006) revealing that it adopts a U shape structure, with the catalytic *a* and *a'* domains facing each other. NMR and x-ray crystallography has further demonstrated that the *b'* domain contains the chaperone activity responsible for binding ligands and protein substrates in its hydrophobic pocket (Denisov et al., 2009).

The CGHC motif modulates the overall reduction potential of PDI and thus it regulates the catalytic ability of the active site cysteines to actively oxidize or reduce disulphide bonds (Chivers et al., 1997). The reduction potential of PDI is −180 mV, higher than other PDI family members, thus making it a strong oxidizing agent. The individual *a* and *a'* domains have similar oxidizing ability but conversely they have low isomerase activity (Darby et al., 1998). The *b'* domain is the main site for binding misfolded protein substrates but the other domains also assist in this process (Klappa et al., 1998). The catalytic domains can only catalyze basic disulphide exchange and all the domains are required to isomerize a protein substrate that has undergone conformational changes (Darby et al., 1996). Deletion of the C-terminal residues of PDI results in deactivation of its chaperone-like activity and its peptide binding ability, but this does not affect its catalytic activity in disulphide bond formation (Dai and Wang, 1997).

Although it is implied that all PDI family members possess the ability to rearrange disulphide bonds, only a few members have actually been demonstrated to perform these activities and the rest are linked to the family through evolution rather than function (Galligan and Petersen, 2012). The most commonly studied members of the PDI family after PDI are ERp57, ERp72, ERp29, ERp44, and PDIA2 (Appenzeller-Herzog and Ellgaard, 2008). There appears to be an interplay of functions amongst the PDI family and some family members are able to recompense for each other. For example, ERp72 is known to compensate for ERp57 deficiency, where it can assist in folding specific proteins (Solda et al., 2006). Certain protein substrates also appear to interact simultaneously with PDI and its family members. ERp57 and PDI engage simultaneously in forming mixed disulphides with thyroglobulin during the production and isomerization of new disulphide bonds. In addition both ERp57 and PDI are released from thyroglobulin when it dissociates from the ER (Di Jeso et al., 2005). Transferrin also requires both PDI and ERp57 for optimal folding. Furthermore, depletion of both PDI and ERp57 leads to generalized protein misfolding, impaired export from the ER, and degradation in human hepatoma cells, implying that these proteins function together (Rutkevich et al., 2010). Functional analysis in yeast revealed that ERp46 substitutes for PDI-mediated disulphide bond formation *in vivo* (Knoblach et al., 2003). However, PDI itself plays a key role in oxidative protein folding and no other family member can entirely compensate for its loss (Rutkevich et al., 2010).

There is also evidence that PDI family members dimerise and that this property is involved in its function. PDI was recently shown to form disulphide-independent dimers *in vivo* suggesting that dimerization may control efficient protein folding in the ER (Bastos-Aristizabal et al., 2014). This may be achieved by generating a reserve of inactive protein that allows the ER to respond competently to an abrupt increase in substrate availability (Bastos-Aristizabal et al., 2014). PDI family member ERp29 also dimerises, and it acts as an escort protein in the binding of thyroglobulin in the ER (Rainey-Barger et al., 2007). It has been suggested that the formation of a dimer of PDIA2, which is mediated through glycosylation (Walker et al., 2013), is increased under conditions of oxidative stress, and this dimer has increased chaperone activity compared to the monomeric form (Fu and Zhu, 2009). Several excellent recent reviews have discussed the structural aspects of the PDI family in more detail and the reader is directed to these for further information (Hatahet and Ruddock, 2009; Kozlov et al., 2010; Galligan and Petersen, 2012). This review will focus on recent advances made into the functional roles of PDI.

## Functions of PDI

PDI is found in all eukaryotic organisms, whereas in prokaryotes a related homolog, Dsb, performs similar functions in facilitating protein folding (Inaba, 2009). The importance of PDI in cellular function was first realized in yeast, where PDI was found to be essential for cellular viability (LaMantia et al., 1991). To date, no viable PDI knockout strain has been reported in rodents, further emphasizing the importance of PDI in normal cellular physiology (Hatahet and Ruddock, 2009). The disulphide interchange and chaperone functions of PDI are well documented and will be summarized briefly below. Emerging evidence describing novel functions for PDI will then be described.

### PDI is a chaperone present in the ER

PDI has the ability to distinguish between partially folded, unfolded, and properly folded protein substrates, and it has a higher affinity to bind to misfolded proteins rather than native proteins through hydrophobic interactions (Klappa et al., 1997). These properties, together with its conformational flexibility, make PDI a highly effective chaperone (Irvine et al., 2014). PDI binds to a large number of protein substrates in the ER, although it is difficult to isolate and identify the individual substrates *in vivo*. Several methods are used to measure the chaperone activity of PDI *in vitro*. The rate of protein aggregation is assessed using protein substrates that do not possess cysteine residues, including GAPDH (Cai et al., 1994), rhodanese (Song and Wang, 1995), citrate synthase, alcohol dehydrogenase (Primm et al., 1996), or GFP, which on interaction with PDI causes increased fluorescent intensity as it folds into its native conformation (Mares et al., 2011).

A major function of PDI is a chaperone upregulated during ER stress. Accumulation of misfolded proteins within the ER activates the unfolded protein response (UPR). The UPR aims to reduce the load of unfolded proteins by increasing the curvature of ER, reducing protein synthesis, and by the induction of PDI and other chaperones to further increase the protein folding capacity (Hetz and Mollereau, 2014). This is achieved by activation of sensor ER proteins inositol requiring enzyme-1(IRE-1), protein kinase RNA like ER kinase (PERK), and activating transcription factor kinase 6 (ATF6), which subsequently activate UPR signaling pathways [detailed in (Sovolyova et al., 2014)]. While initially protective, prolonged UPR causes apoptosis (Schroder and Kaufman, 2005).

PDI facilitates the degradation of misfolded proteins *via* ER association degradation (ERAD) by translocation of these proteins from the ER to the cytoplasm, for subsequent degradation by the ubiquitin protease system. (Molinari et al., 2002; Lee et al., 2010b). It also helps in protein quality control by retaining unassembled procollagen until the correct native structure is achieved (Bottomley et al., 2001).

Other specific functions involving the chaperone activity of PDI have been described, including maintenance of the active conformation of the β subunit of collagen prolyl 4-hydroxylase (Vuori et al., 1992) and stabilization of the major histocompatibility complex's (MHC) class 1 peptide loading complex (PLC) that mediates MHC class 1 folding. Interestingly, PDI exhibits both chaperone and anti-chaperone activity depending upon its initial concentration. When PDI's chaperone activity is dominant, virtually all of the substrate protein is correctly folded. However, at low concentrations, PD1 promotes intermolecular disulphide crosslinking of substrates into large inactive aggregates via anti-chaperone activity (Puig and Gilbert, 1994).

### Redox regulation of PDI

**Figure 2:**
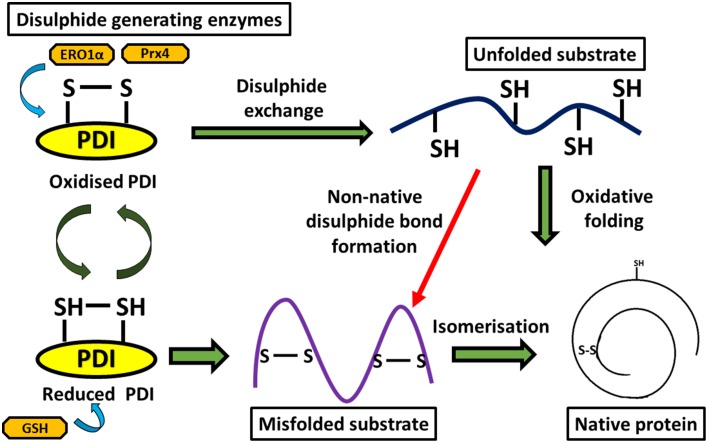
**Diagram representing disulphide bond formation in the eukaryotic ER and redox reactions involving PDI**. Oxidative folding of PDI leads to disulphide bond formation in native protein substrates. Reduced PDI facilitates isomerization of non-native bonds in protein substrates.

Multiple studies have suggested that the disulphide interchange enzymatic activity of PDI is more important for its function than its chaperone activity. When its catalytic cysteines are reduced, PDI is able to react with non-native disulphides of substrate proteins to form a mixed disulphide complex. PDI then catalyzes the rearrangement of incorrectly formed disulphide bonds via isomerization reactions. This takes place with cycles of reduction (breaking of non-native disulphide bonds) and oxidation (to introduce correct pairing of cysteines) to eventually form the native disulphide bonds (Schwaller et al., 2003). The tripeptide glutathione constitutes the cellular redox buffer that maintains the redox environment of the ER (Hwang et al., 1992). After PDI has oxidized substrate proteins, it then has to be oxidized itself to complete the catalytic cycle. This function is carried out by a number of proteins including FAD binding oxidases, Ero1α, oxidized glutathione, glutathione peroxidase 7, glutathione peroxidase 8 or quiescin sulfhydryl oxidase (Wilkinson and Gilbert, 2004) (Figure 2). Interestingly, the chaperone activity of PDI is regulated by the redox state of its oxidized and reduced forms (Wang et al., 2013), suggesting a link between the two separate functions of PDI. Redox regulation of PDI can be examined experimentally *in vitro* using scrambled RNAse, ribonuclease and bovine pancreatic trypsin inhibitor (Darby et al., 1998; Xiao et al., 2001).

The *in vivo* redox state of PDI is complex and determined by numerous factors including the reduction potential of PDI, the glutathione redox state in the ER, and the potential reductase activity of the substrate and its availability. However, redox conditions can have a major impact on the functions of PDI. For example, PDI regulates the organization of the cytoskeleton by forming a disulphide bond to Cys 374 of β-actin *via* a redox dependent mechanism (Sobierajska et al., 2014).

## PDI in disease states

Recent studies provide compelling evidence for a role for PDI in both the physiology and pathophysiology of disease states including diabetes (Grek and Townsend, 2014), cardiovascular diseases (Khan and Mutus, 2014), cancer (Xu et al., 2014), neurodegenerative conditions (Andreu et al., 2012) and the entry of pathogens in infectious diseases (Benham, 2012). However, precise roles for PDI in these diseases have not yet been elucidated. PDI is upregulated in various tissues during disease and surprisingly, both protective and detrimental effects have been described. These effects relate to either a loss of its normal protective function in some situations, or gain of toxic function in others. While the association between the PDI family and human disease states still requires further validation, current improvements in our understanding of the functional roles of PDI provide new insights into the physiological contribution of PDI *in vivo*.

### PDI in cancer

PDI is highly expressed and up-regulated in numerous cancer cell types, including kidney, lung, brain, ovarian, melanoma, prostrate, and male germ cell tumors (Xu et al., 2014). Also, lower levels of PDI are associated with a higher survival rate in patients with breast cancer and glioblastoma (Thongwatchara et al., 2011), suggesting that PDI promotes the survival of cancer cells. Consistent with this notion, knockdown of PDI induces cytotoxicity in human breast cancer and neuroblastoma cell lines due to caspase activation (Hashida et al., 2011). Suppression of apoptosis by PDI has been proposed as mechanism for tumor growth and metastasis. Over-expression of PDI may therefore serve as a diagnostic marker for cancer, as suggested for glial cell cancer (Goplen et al., 2006), colorectal cancer (Ataman-Onal et al., 2013), and breast cancer (Thongwatchara et al., 2011). Cell surface PDI is also associated with cancer progression and administering of anti-PDI monoclonal antibodies inhibits the invasion of glioma cells (Goplen et al., 2006).

As increasing evidence suggests that PDI supports the survival and progression of various cancers, inhibitors of PDI may therefore have a therapeutic role against cancer progression (Xu et al., 2014). A synthesized series of PACMA (propynoic acid carbamoyl methyl amides) compounds demonstrated anticancer activity in human ovarian cancer *in vitro* and *in vivo* by a mechanism involving inhibition of PDI (Xu et al., 2012). Bacitracin, a pharmacological inhibitor of PDI, reduced the *in vitro* migration and invasion of human brain glial cells (Goplen et al., 2006). However, the specificity of bacitracin as an inhibitor of PDI has recently been questioned (Karala and Ruddock, 2010). Small-molecule inhibitors of PDI which bind to the CGHC active site may also have potential for improving the efficacy of chemotherapy in melanoma, as inhibition of PDI function proliferates apoptosis (Lovat et al., 2008). However, the effect of PDI in supporting tumor survival is based on the specific type of cancer and may be cell type dependent. Hence it is important to recognize the specific type of cancer cell for future applications in cancer therapy.

### PDI in neurodegenerative disorders

Neurodegenerative diseases are also known as protein misfolding disorders due to their characteristic property of accumulating insoluble ubiquitinated aggregated proteins within affected tissues. Protein misfolding within the ER triggers ER stress, and hence up-regulation of PDI, and ER stress is increasingly implicated in these diseases (Hetz and Mollereau, 2014). Most studies suggest that the induction of PDI during ER stress in neurodegenerative diseases reduces the load of misfolded proteins, and is therefore protective thus restoring proteostasis and increasing neuronal viability.

PDI is upregulated in dopaminergic neurons and Lewy bodies of patients with Parkinson's disease. Similarly PDI reduces aggregation of the Parkinson's disease-associated protein synphilin-1 in neuroblastoma cells, an activity which relies on the presence of the CGHC active site motif (Uehara et al., 2006). Similarly, PDI also prevents aggregation of another Parkinson's associated protein, α-synuclein, in cell-free *in vitro* systems (Cheng et al., 2010). PDI also co-localizes with neurofibrillary tangles in Alzheimer's disease patient brain tissue, and is upregulated in brains of Alzheimer's rodent models (Lee et al., 2010a) implying a role in refolding misfolded proteins in these conditions. Consistent with this notion, ERp57 is present in CSF, where it binds and reduces aggregation of β-amyloid peptides (Erickson et al., 2005). Furthermore, PDI is upregulated in response to hypoxia in the brain and PDI prevents neuronal and cardiomyocyte apoptosis, triggered by hypoxia-ischaemia in cell culture and in rodent models, by decreasing protein misfolding (Tanaka et al., 2000). In prion disorders, Wang and group suggested a pleiotropic role of PDI in the cellular management of misfolded prion protein (Wang et al., 2012b) because PDI and ERp57 are protective against prion induced toxicity *in vitro* (Hetz et al., 2005) and inhibition of PDI increases the production of misfolded prion proteins (Watts et al., 2009).

An important role for PDI has been implicated in amyotrophic lateral sclerosis (ALS). PDI is up-regulated and recruited to misfolded protein aggregates in sporadic human ALS (Atkin et al., 2008). PDI is also up-regulated in lumbar spinal cords from transgenic SOD1^G93A^ mice, the most widely used animal disease model (Atkin et al., 2006). Furthermore, over-expression of PDI is protective against the formation of mutant SOD1 inclusions and ER stress, whereas knockdown of PDI using siRNA increases mutant SOD1 aggregation and inclusion formation (Walker et al., 2010). Similarly, a small molecule mimic of PDI reduces mutant SOD1 aggregation *in vitro* (Walker et al., 2010). Endogenous PDI co-localizes with mutant superoxide dismutase 1 (SOD1) (Atkin et al., 2006), TAR DNA-binding protein-43 (TDP-43) (Honjo et al., 2011), vesicle associated protein B (VAPB) (Tsuda et al., 2008), and Fused in Sarcoma (FUS) (Farg et al., 2012) in neuronal cells. PDI and ERp57 were identified as potential biomarkers for ALS using peripheral blood mononuclear cells (Nardo et al., 2011) and mutations in intronic variants of PDI are predicted to be a risk factor in ALS (Kwok et al., 2013).

There is also evidence that the cellular location of PDI is linked to disease outcomes in ALS. PDI is redistributed away from the ER via a reticulon-dependent process in transgenic SOD1^G93A^ mice (Yang et al., 2009). The reticulon family of proteins function in maintaining the curvature of ER and several members of this family modulate the re-distribution of PDI away from the ER when overexpressed (Bernardoni et al., 2013). Furthermore, deletion of reticulon 4a,b enhances disease progression in SOD1^G93A^ mice (Yang et al., 2009), highlighting the importance of a non-ER location of PDI in ALS.

### Roles of PDI in cardiovascular disease

Both beneficial and harmful roles for PDI in cardiovascular disease have been proposed. PDI prevents protein misfolding in the myocardium during ischemic myocardial injury (Toldo et al., 2011). PDI is also up-regulated in hypoxia induced in myocardial capillary endothelial cells (Tian et al., 2009) and this is linked to significant decreases in the rate of cardiomyocyte apoptosis in murine models (Severino et al., 2007). Similarly, PDI is also involved in endothelial cell endurance (Severino et al., 2007) and it is required for platelet derived growth factor (PDGF)-induced vascular smooth muscle cell migration (Primm and Gilbert, 2001) which is an important therapeutic target in atherosclerosis (Pescatore et al., 2012). Furthermore, increased expression of PDI is protective against endothelial cellular migration, adhesion, and tubular formation in mice suggesting an important role for PDI in angiogenesis (Tian et al., 2009). Hence these studies raise the possibility that upregulating PDI has possible future pharmacological applications in treating ischemic cardiomyopathy (Severino et al., 2007).

Diabetes is associated with coronary artery disease and an increased risk of heart failure, and PDI function is impaired in mouse models of diabetes. This may be due to alterations in its oxidoreductive state (Toldo et al., 2011). Reduced PDI has been detected in the diabetic heart after ischemia, which could explain why PDI is not protective in diabetes (Toldo et al., 2011).

However, in contrast to these protective functions, PDI has also been implicated in detrimental activities in cardiovascular diseases. Over-expression of PDI in myocytes attenuates the levels of misfolded pro-insulin while decreasing glucose stimulated insulin secretion, thereby inducing ER stress and apoptosis (Zhang et al., 2009). PDI on the surface of platelets plays an important role in thrombus formation and it is vital for the aggregation of platelets (Kim et al., 2013). Similarly, PDI is also present on at the surface of human B-lymphocytes where it has a putative role in regulating leukocyte adhesion (Bennett et al., 2000). PDI has also been implicated in platelet integrin function, tissue-factor activation, and in mice, it accumulates during fibrin and thrombus formation at sites of vascular injury (Jurk et al., 2011). PDI inhibition prevents both platelet accumulation and fibrin generation during thrombus formation (Jasuja et al., 2012). Therefore, inhibition of PDI could prevent thrombosis in coronary artery disease, suggesting that PDI inhibitors have potential as antithrombotic agents (Jasuja et al., 2012).

### PDI mediates pathogen entry in infectious diseases

PDI is also implicated in mediating the entry of pathogens during infectious disease. Over-expression of PDI increases the fusion of viral membranes, leading to internalization of HIV-1 (Auwerx et al., 2009). Similarly, cell surface PDI facilitates infection of HeLa cells by mouse polyoma virus (Gilbert et al., 2006), and it also mediates the entry of cholera toxin (Stolf et al., 2011). The chaperone activity of PDI is important for folding cholera toxin subunit A1 and reducing its aggregation (Taylor et al., 2011). However, cholera intoxication is a redox dependent process. The oxidized form of PDI mediates translocation of cholera toxin into the host cell cytoplasm (Tsai et al., 2001) whereas the reduced form of PDI leads to its unfolding.

## Post translational modification of PDI

Redox-dependent post translational modifications of PDI are also linked to disease states. Due to cellular conditions, high levels of reactive nitrogen species (RNS), hydrogen peroxide and reactive oxygen species (ROS) can accumulate in cells, inducing nitrosative or oxidative stress. Nitrosative stress can lead to post translation modification of PDI by the addition of NO to active site cysteine residues, resulting in S-nitrosylation. S-nitrosylation of proteins under pathological conditions is an abnormal, irreversible process that is linked to protein misfolding, ER stress and apoptosis. Furthermore, proteins resident in the ER are particularly vulnerable to post translation modification due to the presence of critical redox regulated cysteines. Since PDI is the major enzyme responsible for modification of protein disulphide bonds, the loss of function of PDI could increase cellular protein misfolding and thus increase ER stress. S-nitrosylation of PDI inhibits its normal enzymatic activity and hence the beneficial effects of PDI, and S-nitrosylated PDI has been detected in several neurodegenerative diseases (Nakamura and Lipton, 2011; Chen et al., 2012). S-nitrosylation reduces both its chaperone and isomerase activity (Uehara et al., 2006).

S-nitrosylation of PDI has been detected in postmortem brain tissues of patients with Alzheimer's disease, Parkinson's disease (Uehara et al., 2006) and in lumbar spinal cord tissues of ALS patients and transgenic SOD1^G93A^ mice (Walker et al., 2010). S-nitrosylation has also been reported in prion disease models using brain tissues of scrapie-263K-infected hamsters (Wang et al., 2012b). Exposure of cultured neurons to N-methyl-D-aspartate receptor (NMDA), leading to calcium influx and nitric oxide production, also resulted in the S-nitrosylation of PDI (Forrester et al., 2006). S-nitrosylated PDI (SNO-PDI) increases the levels of polyubiquitinated proteins and triggers cell death, and it is also associated with hyper-activation of NMDA (Forrester et al., 2006) and inhibition of mitochondria, leading to the generation of ROS and nitric oxide (Halloran et al., 2013). SNO-PDI accentuates the misfolding of synphilin in Parkinson disease (Forrester et al., 2006) and S-nitrosylation also increases mutant SOD1 aggregation via incorrect disulphide cross-linking of the immature, misfolded mutant SOD1, leading to neuronal cell death (Jeon et al., 2014).

As well as S-nitrosylation, other aberrant post-translational modifications of PDI have been described, including carbonylation and S-glutathionylation. Oxidized low density lipoproteins induce carbonylation, which disrupts the catalytic activity of PDI, inducing ER stress and apoptosis in vascular cells (Muller et al., 2013). Furthermore, carbonylated PDI detected in the lipid rich atherosclerotic region of human endothelial cells activates CHOP and XBP1 and induces apoptosis (Muller et al., 2013). S-glutathionylation is induced by reactive oxygen or nitrogen species and it results in formation of a disulphide bond between GSH and a cysteine residue of another protein (Xiong et al., 2011). S-glutathionylation, leading to increased protein misfolding and enhancement of the UPR (Townsend et al., 2009), has been detected primarily in relation to cancer. S-glutathionylation of PDI obliterates estrogen receptor α stability in breast cancer cells, which prevents binding of PDI to the receptor. This subsequently leads to dysregulation in ERα signaling (Xiong et al., 2012), and cell death via UPR induction (Xiong et al., 2012). S-glutathionylation also reduces the isomerase activity of PDI in ovarian cancer cells and human leukemia cells and it also decreases chaperone activity. In cultured astrocytes after cerebral ischemic reperfusion, SNO-PDI increases the levels of ubiquitinated aggregates that co-localize with SOD1 (Chen et al., 2012). These modifications can further attenuate UPR and cause neuronal cell death. Hence, aberrant modifications of PDI lead directly to harmful effects as well as loss of the normally protective properties of PDI.

### PDI causes oxidative stress

Recent evidence implicates PDI in increasing the levels of ROS, thus directly inducing oxidative stress and apoptosis *via* its chaperone activity rather than the disulphide interchange activity (Fernandes et al., 2009). Similarly, only oxidized PDI triggers the production of ROS, whereas reduced PDI inhibits the production of ROS (Paes et al., 2011). PDI associates with the NAPDH peroxidase complex (Nox), a major source of ROS, where it stabilizes and associates with the oxidase subunit of Nox in vascular smooth muscle cells (Janiszewski et al., 2005). Similar effects are observed in macrophages and murine microglial cells, where PDI interacts with Nox and increases the levels of ROS (Fernandes et al., 2009). PDI also activates the transcription factors NF-kB and AP-1, thus promoting their binding to DNA (Clive and Greene, 1996). PDI is also a major catalyst of trans-nitrosation reactions, mediating nitric oxide internalization from extracellular S-nitrosothiols (Zai et al., 1999), thus further promoting the production of SNO proteins (Ramachandran et al., 2001).

### PDI causes apoptosis

Whilst SNO-PDI is implicated in triggering apoptosis, recent studies have revealed a direct role for unmodified PDI in apoptosis. In rat models of Huntington and Alzheimer's disease, PDI accumulation at the ER-mitochondrial junction triggers apoptosis *via* mitochondrial outer membrane permeabilisation (Hoffstrom et al., 2010). This effect is specific for the accumulation of misfolded proteins, but not other triggers of apoptosis, suggesting a specific role for pro-apoptotic PDI in neurodegenerative disease. Inhibitors of PDI including hypotaurine, thiomuscimol, and shRNA that inhibited the activity of PDI, were found to suppress the toxicity associated with misfolded Huntingtin and β-amyloid proteins.

## Summary

PDI is an important cellular protein given its abundance, multiple biological functions, versatile redox behavior, interaction with other proteins and its implied role in various diseases. However, many issues remain unresolved that warrant further investigation, in particular the role of PDI in non-ER sub-cellular locations, and substrate specificity of the PDI family members. In future studies it will be important to replicate the precise functions of PDI in the ER and other cellular locations, separately from roles ascribed *in vitro*, before its normal cellular roles are fully understood.

**Figure 3:**
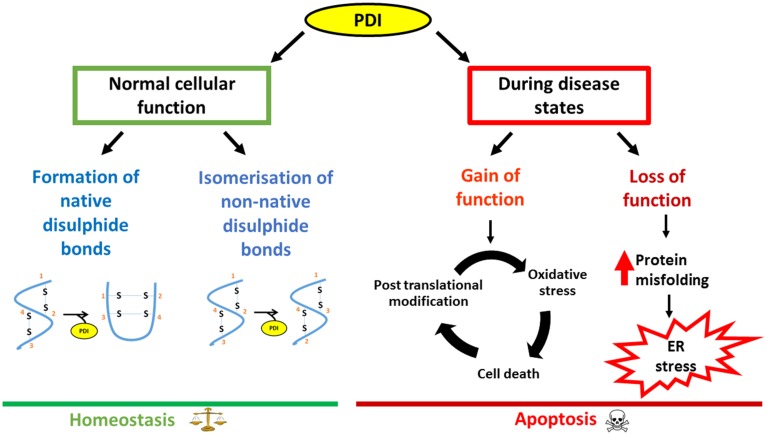
**Schematic diagram outlining the dual nature of PDI, focusing on neurodegenerative disorders as an example**. Under normal conditions, PDI reduces the load of misfolded proteins either by its chaperone activity or by isomerization of non-native bonds. However, during disease states, loss of the normal protective function of PDI as well as the gain of additional, toxic functions, leads to PDI becoming apoptotic, thus contributing to pathology.

PDI performs an impressive array of cellular functions and the up-regulation of PDI is a cellular defensive mechanism to restore proteostasis. However, despite this up-regulation, the functional properties of PDI can become abrogated due to aberrant post translational modifications. This is of particular relevance in neurodegenerative diseases where disruption to redox regulation is implicated (Parakh et al., 2013). Furthermore, neurons are particularly susceptible to ROS/RNS damage due to their high oxygen demand and a lower availability of antioxidants. Recent evidence implicates PDI as a trigger for apoptosis specifically in relation to the accumulation of misfolded proteins. PDI may therefore act as a regulatory switch, in which PDI is initially is protective against protein misfolding and aggregation. However, in response to an unknown trigger PDI subsequently becomes apoptotic when proteostasis cannot otherwise be resolved (Figure 3). Therefore aberrant post translational modifications together with the pro-apoptotic function of PDI could further accentuate the adverse effects of PDI.

**Figure 4:**
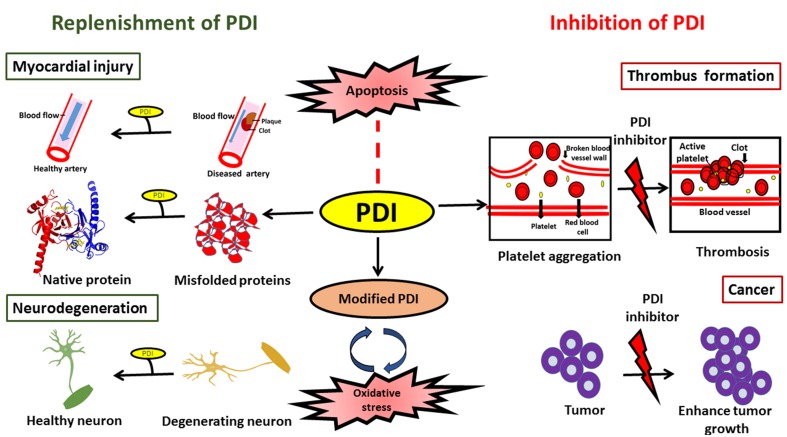
**Schematic diagram illustrating possible therapeutic applications to modulate PDI function**.

In conclusion, PDI is an efficient catalyst and protein chaperone. It has the ability to restore proteostasis by catalyzing the efficient folding of newly synthesized proteins, and it plays an important role in protein quality control and ERAD by reducing the burden of misfolded proteins, thus inhibiting abnormal protein aggregation. The protective or harmful functions of PDI may be modulated by the subcellular location of PDI, levels of ER stress and the redox environment. While further investigations are clearly needed in this area, PDI has the potential to be exploited therapeutically in a variety of diseases. However, specific approaches depending on the disease in question will be required. In neurodegenerative conditions, elevation of the levels of total PDI, with the aim of restoring PDI function to reduce protein misfolding, could be an effective therapeutic approach. However, in contrast, reduction of the levels of PDI might be an appropriate strategy in cancer or cardiovascular diseases (Figure 4). Similarly, reducing the levels of aberrantly modified PDI might also be necessary in neurodegeneration in order to defend against the pro-apoptotic properties of PDI. At the cellular level there are important unanswered questions that need addressing, before the therapeutic applications of PDI can become realized in the future.

### Conflict of interest statement

The authors declare that the research was conducted in the absence of any commercial or financial relationships that could be construed as a potential conflict of interest.
